# Energy Characteristics of a Bidirectional Axial-Flow Pump with Two Impeller Airfoils Based on Entropy Production Analysis

**DOI:** 10.3390/e24070962

**Published:** 2022-07-11

**Authors:** Fan Meng, Yanjun Li

**Affiliations:** 1 Research Center of Fluid Machinery Engineering and Technology, Jiangsu University, Zhenjiang 212013, China; lyj782900@ujs.edu.cn; 2 Wenling Fluid Machinery Technology Institute of Jiangsu University, Wenling 317525, China

**Keywords:** bidirectional axial-flow pump, S-shaped impeller, arc-shaped impeller, entropy production, total entropy production rate

## Abstract

This research sought to determine the spatial distribution of hydraulic losses for a bidirectional axial-flow pump with arc- and S-shaped impellers. The unsteady Reynolds time-averaged Stokes (URANS) approach with the SST k-omega model was used to predict the internal flow field. The total entropy production (TEP) and total entropy production rate (TEPR) were used to evaluate the overall and local hydraulic losses. The results show that the distribution of TEP and TEPR was similar for both impeller cases. Under a forward condition, TEP mainly comes from the impeller and elbow pipe. The high TEPR inside the impeller can be found near the shroud, and it shifts from the leading edge to the trailing edge with an increase in the flow rate due to the decline in the attack angle. The high TEPR inside the elbow pipe can be seen near the inlet, and the area shrinks with an increase in the flow rate caused by a reduction in the velocity circulation. Under the reverse condition, TEP mainly comes from the impeller and the straight pipe. The TEPR of the region near the shroud is obviously higher than for other regions, and the area of high TEPR near the suction side shrinks with an increase in the flow rate. The high TEPR of the straight pipe can be found near the inlet, and declines in the flow direction. These results provide a theoretical reference for future work to optimize the design of the bidirectional axial-flow pump.

## 1. Introduction

The bidirectional axial-flow pump can achieve two-way pumping by directly changing the rotation direction of the motor, which has the advantages of low civil engineering costs, stable operation, and easy maintenance. Therefore, it is becoming more widely used in agricultural engineering for both drainage and irrigation requirements [1,2]. When the conventional one-way axial-flow impeller rotates in the opposite direction, the camber of the airfoil is negative, which leads to strong flow separation near the blade surface [3,4]. The large-scale flow separation vortices near the blade surface collide with the main stream, which results in serious cavitation, vibration, and an obvious decline in hydraulic performance [5]. To balance the forward and reverse pumps’ performance, the airfoils of the impeller in the bidirectional axial-flow pump were designed to be arc- and S-shaped [6]. Due to the special airfoils, the hydraulic design theory of the traditional axial-flow impeller is not applicable. The current lack of mature theoretical guidance on this airfoil design hinders the further improvement of the hydraulic performance of the bidirectional axial-flow pump.

In benefiting from the development of computational fluid dynamics (CFD) technology [7], the hydraulic performance [8,9] and internal flow structure [10,11] of an axial-flow pump can be predicted accurately, which can provide a reference for hydraulic design. For instance, Shi [12,13] explored the effect of backflow clearance on the internal unstable flow structure and pressure pulsation characteristics of an axial-flow pump. The researcher asserted that leakage flow caused by backflow clearance reduced the axial velocity at the impeller inlet, resulting in a reduction in the hydraulic performance and high-efficiency operating range. Meanwhile, Feng [14] investigated the influence of the tip clearance radius on the internal pressure pulsation characteristics of an axial-flow pump. The results showed that the intensity of the tip clearance leakage vortex increases with an increase in the tip clearance radius, enhancing the pressure fluctuation intensity on the blade. Elsewhere, Fei [15] studied the effect of the blade angle on the hydraulic performance and tip leakage vortex (TLV). The results showed that the pump head, best efficiency point, and TLV intensity increase with an increase in the blade angle. Beyond this, Yang [16] analyzed the influence of the deflection angle on the internal flow characteristics in the inlet passage of an axial-flow pump. He found that the inflow pattern gradually worsened with an increase in the deflection angle, and the dominant frequency amplitude of the monitoring point under the deflection inflow was significantly higher than that under the vertical inflow. However, analysis results for the velocity and pressure fields in the axial-flow pump alone cannot directly determine the hydraulic losses. As the impeller airfoil is affected by numerous control parameters, it is necessary to determine the spatial distribution of hydraulic losses in a bidirectional axial-flow pump to optimize the control parameters efficiently.

Thanks to the continuous improvement of entropy production theory by Herwig and Knock [17,18,19], the hydraulic losses in rotating machinery [20,21] can be quantitatively evaluated based on the entropy production, which can be calculated in the CFD post-processing. Many scholars have used entropy production theory to visually analyze the distribution of hydraulic losses in pumps. Guan [22], for instance, studied the effects of flow rates on the distribution of entropy production in a double-suction centrifugal pump. The results showed that the distribution of entropy production was mainly affected by the main flow characteristic. The vortex size decreased with an increase in the flow rate, which led to a decline in the entropy production rate. Meanwhile, Zhang [23] compared the distributions of entropy production in a side-channel pump, and found that the entropy production in the inner radius of the impeller passage was higher than for the outer radius. Elsewhere, Ji [24] and Shen [25] evaluated the hydraulic losses in a mixed-flow pump and an axial-flow pump, respectively, based on entropy production. They proposed that the intensity of the tip leakage flow rose with an increase in the tip clearance radius under the design condition, which led to an increase in the entropy production in the impeller. When compared with the traditional axial-flow pump, the hydraulic design of the bidirectional axial-flow pump differs in that it needs to balance the forward and reverse hydraulic performance. Under the reverse condition, the inlet anti-arch guide vanes distort the impeller inflow and produce great hydraulic losses. Accordingly, the hydraulic design of the bidirectional axial-flow pump is complex, and the high hydraulic losses need to be located to provide a theoretical reference. No existing public literature could be found that analyzed the distribution of the entropy generation in the bidirectional axial-flow pump.

In this study, the unsteady Reynolds time-averaged Stokes (URANS) approach was used to predict the internal flow structures of arc- and S-shaped bidirectional axial-flow pumps, and the calculated results were validated by external characteristic test data. Based on entropy production theory, the total entropy production in each hydraulic component was determined under different flow rates. The spatial distributions of entropy production rates in the impellers and guide vanes were analyzed, and combined with the velocity field. The results provide useful suggestions for how to optimize the design of arc- and S-shaped bidirectional axial-flow pumps.

## 2. Numerical Simulation

### 2.1. Three Models and Meshes

**Figure 1:**
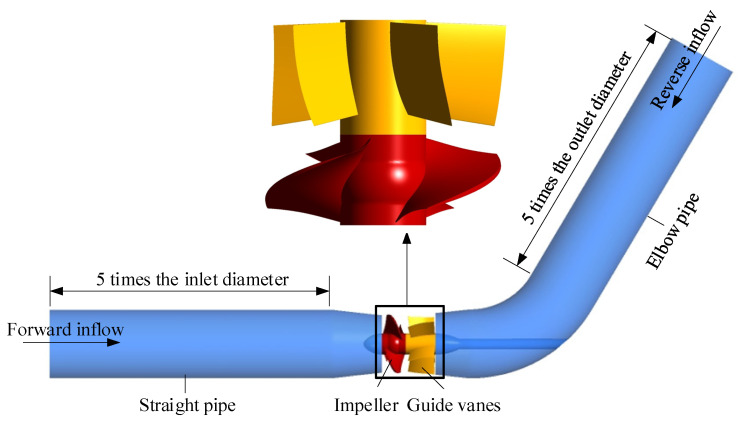
Three models of the bidirectional axial-flow pump.

**Figure 2:**
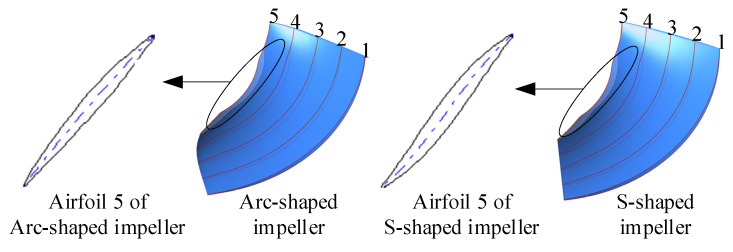
Comparison of arc- and S-shaped impeller airfoils.

In this study, a bidirectional axial-flow pump with an arc-shaped impeller was used, as shown in Figure 1. Except for the impeller, the two kinds of axial-flow pumps were the same in that they contained the same straight pipe, guide vanes, and elbow pipe. Figure 2 shows a comparison of the arc- and S-shaped impellers both based on five airfoils. The blade number, diameter, and tip clearance for the two impellers were 3, 300, and 0.2 mm, respectively. The blade number and hub diameter of the guide vanes were 5 and 120 mm, respectively. Further main design parameters for the two bidirectional axial-flow pumps are listed in Table 1.

**Figure 3:**
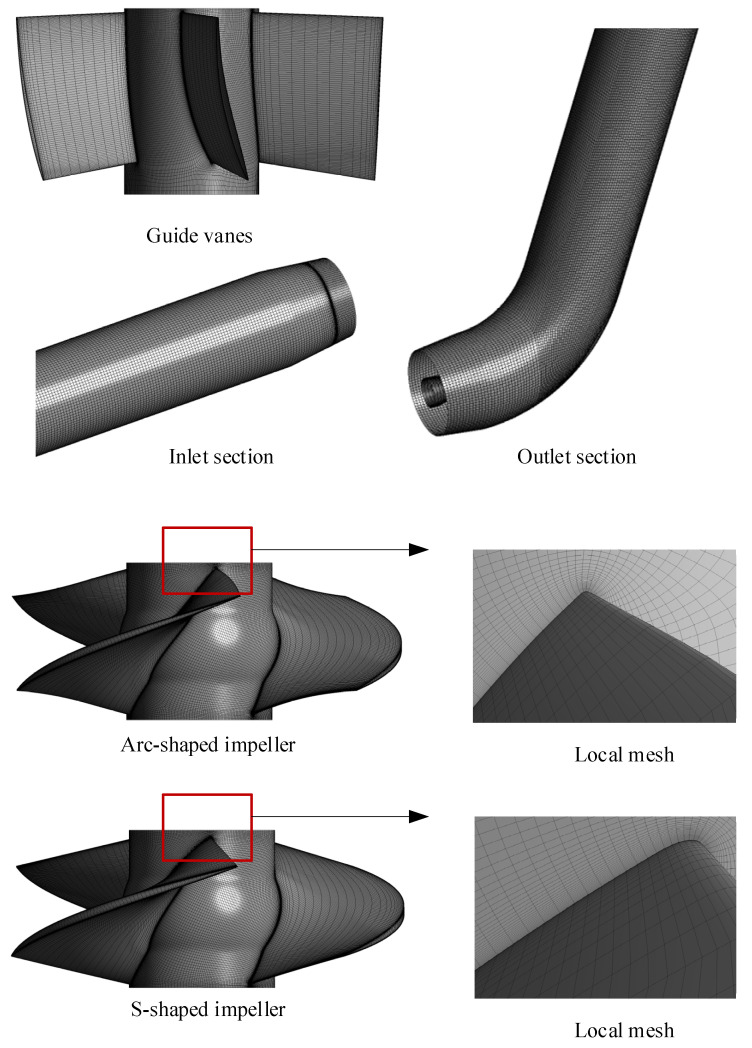
Mesh of each hydraulic component of the bidirectional axial-flow pump.

**Figure 4:**
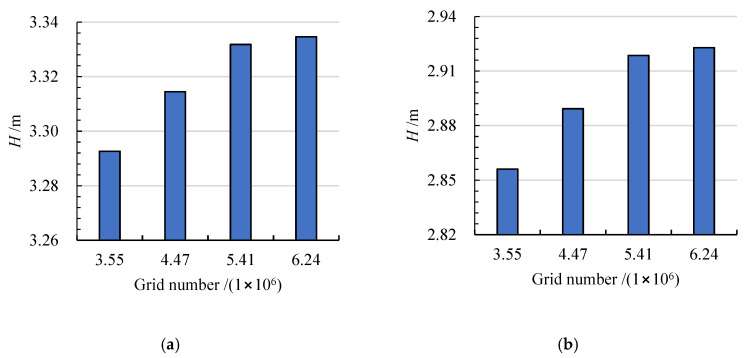
Grid independence analysis of the arc-shaped impeller case under (**a**) forward and (**b**) reverse conditions.

The computational domains of the two bidirectional axial-flow pumps were discretized by a hexahedral mesh. As shown in Figure 3, ICEM CFD was used to generate meshes for the straight and elbow pipes. TurboGrid was applied to create meshes for the arc-shaped impeller, S-shaped impeller and guide vanes. The average Y+ values of the straight pipe, arc-shaped impeller, S-shaped impeller, guide vanes, and elbow pipe were 27.9, 23.3, 24.6, 11.2, and 33.3, respectively, which met the requirements of the k−ω SST turbulence model [26]. The grid numbers of the two bidirectional axial-flow pumps were determined by grid independence analysis, as shown in Figure 4. Since the total grid nodes exceeded 5.41 million, the forward and reverse design heads’ relative growth rates for the arc-shaped impeller case were less than 0.02% and 0.17%, respectively. Accordingly, the number of grid nodes of the straight pipe, arc-shaped impeller, guide vanes, and elbow pipe were determined to be 909,706, 2,079,720, 1,478,235, and 950,172, respectively. To ensure that the number of nodes would not affect the comparison of the two impeller cases, the number of grid nodes for the S-shaped impeller was calculated as 2,279,160.

### 2.2. Boundary Condition

In this study, the governing equation of the computational domain was an RANS equation, as follows [27]:

(1) $$\frac{\partial {\bar{v}}_{j}}{\partial x_{j}}=0$$

(2) $$\frac{\partial (\rho {\bar{v}}_{i})}{\partial t}+\frac{\partial (\rho {\bar{v}}_{i}{\bar{v}}_{j})}{\partial x_{j}}=-\frac{\partial \bar{p}}{\partial x_{i}}+\frac{\partial }{\partial x_{j}}(\mu \frac{\partial {\bar{v}}_{i}}{\partial x_{j}}-\bar{\rho v_{i}^{\prime }v_{j}^{\prime }})+\rho f_{i}$$

where ρ, $\bar{p}$, and μ are the water density, time-averaged pressure, and dynamic viscosity, respectively, and $\bar{v}$ is the time-averaged velocity. The subscripts i and j represent the *x*, *y*, and *z* directions in the Cartesian coordinate system.

The steady calculation results were used as the initial values of the unsteady calculations, which were completed to predict the internal flow fields of the two bidirectional axial-flow pumps. The inlet and outlet conditions were set as “Mass Flow Rate” [28] and “Opening Pres. And Dirn.” The inlet flow rate was adjusted according to the operating conditions, and the outlet relative pressure was set to 0 Pa. The wall surface roughness scores of the inlet section, impeller, guide vanes, and outlet section were 0.05, 0.0125, 0.0125, and 0.05 mm, respectively. The interface between the stators was set as “None.” The interfaces between the rotor and stator for steady and unsteady calculations were set as “Stage” [29] and “Transient Rotor Stator” [30], respectively. In addition, the time step was 0.00037037 s, i.e., a rotation of 3° per time step [31]. The total computation time was 0.533333 s.

## 3. Entropy Production Theory

Entropy production is a state quantity that can parameterize the transformation of energy within the computational domain. Based on the second law of thermodynamics, the time-averaged transport equation for entropy production can be obtained as follows [18,19]:

(3) $$\rho (\frac{\partial S}{\partial t}+v_{1}\frac{\partial S}{\partial x}+v_{2}\frac{\partial S}{\partial y}+v_{3}\frac{\partial S}{\partial z})=div(\frac{\vec{q}}{T})+\frac{\Phi }{T}+\frac{\Phi_{\theta }}{T^{2}}$$

where S is the specific entropy, T and $\vec{q}$ represent the temperature and the heat flux density vector, respectively, $\frac{\Phi }{T}$ and $\frac{\Phi_{\theta }}{T^{2}}$ represent the entropy production by dissipation and heat transfer, respectively, and $v_{1}$, $v_{2}$, and $v_{3}$ represent the x, y, and z directions of the Cartesian coordinate system, respectively.

During the operation of a bidirectional axial-flow pump, part of the mechanical energy of the motor is irreversibly converted into internal energy due to the existence of working medium viscosity and Reynolds stress, resulting in irreversible hydraulic losses. The working medium is pure water, and there are no chemical reactions leading to significant temperature fluctuations in the bidirectional axial-flow pump. The internal flow field is thus considered to be at a constant temperature and incompressible, and the local hydraulic losses can be evaluated quantitatively by $\frac{\Phi }{T}$.

Since the governing equation of the computational domain is an RANS equation, the transport equation for the entropy production shroud can be time-averaged, so $\frac{\Phi }{T}$ can be calculated in the CFD post-processing. After time-averaging, $\bar{\left(\frac{\Phi }{T}\right)}$ can be divided into the entropy production rate by direct dissipation $\frac{\Phi_{\bar{D}}}{\bar{T}}$ and the entropy production rate by indirect dissipation $\frac{\Phi_{D^{\prime }}}{\bar{T}}$, as follows [17,18]:

(4) $$\bar{\left(\frac{\Phi }{T}\right)}=\frac{\Phi_{\bar{D}}}{\bar{T}}+\frac{\Phi_{D^{\prime }}}{\bar{T}}$$

(5) $$\frac{\Phi_{\bar{D}}}{\bar{T}}=\frac{\mu }{\bar{T}}\left\{2\times \left[{\left(\frac{\partial \bar{v_{1}}}{\partial x}\right)}^{2}+{\left(\frac{\partial \bar{v_{2}}}{\partial y}\right)}^{2}+{\left(\frac{\partial \bar{v_{3}}}{\partial z}\right)}^{2}\right]+{\left(\frac{\partial \bar{v_{1}}}{\partial y}+\frac{\partial \bar{v_{2}}}{\partial x}\right)}^{2}+{\left(\frac{\partial \bar{v_{3}}}{\partial x}+\frac{\partial \bar{v_{1}}}{\partial z}\right)}^{2}+{\left(\frac{\partial \bar{v_{2}}}{\partial z}+\frac{\partial \bar{v_{3}}}{\partial y}\right)}^{2}\right\}$$

(6) $$\frac{\Phi_{D^{\prime }}}{\bar{T}}=\frac{\mu }{\bar{T}}\left\{2\times \left[\bar{{\left(\frac{\partial v_{1}^{\prime }}{\partial x}\right)}^{2}}+\bar{{\left(\frac{\partial v_{2}^{\prime }}{\partial y}\right)}^{2}}+\bar{{\left(\frac{\partial v_{3}^{\prime }}{\partial z}\right)}^{2}}\right]+\bar{{\left(\frac{\partial v_{1}^{\prime }}{\partial y}+\frac{\partial v_{2}^{\prime }}{\partial x}\right)}^{2}}+\bar{{\left(\frac{\partial v_{3}^{\prime }}{\partial x}+\frac{\partial v_{1}^{\prime }}{\partial z}\right)}^{2}}+\bar{{\left(\frac{\partial v_{2}^{\prime }}{\partial z}+\frac{\partial v_{3}^{\prime }}{\partial y}\right)}^{2}}\right\}$$

where the superscripts $\overset{}{¯}$ and $\prime^{}$ represent the time-averaged component and the fluctuation component, respectively. Since the velocity fluctuation component cannot be obtained by solving the URANS equation, Knock [18,19] proposed a model equation to obtain the approximate solution of $\frac{\Phi_{D^{\prime }}}{\bar{T}}$, as follows:

(7) $$\frac{\Phi_{D^{\prime }}}{\bar{T}}=\frac{\rho \varepsilon }{\bar{T}}$$

where ε is the dissipation rate of turbulent kinetic energy. In summary, the overall hydraulic losses of each hydraulic component of a bidirectional axial-flow pump can be characterized by the overall entropy production, which can be obtained by integrating $\frac{\Phi_{\bar{D}}}{\bar{T}}$ and $\frac{\Phi_{D^{\prime }}}{\bar{T}}$.

(8) $$S_{PRO,\bar{D}}=\int_{V}\frac{\Phi_{\bar{D}}}{\bar{T}}dV$$

(9) $$S_{PRO,D^{\prime }}=\int_{V}\frac{\Phi_{D^{\prime }}}{\bar{T}}dV$$

(10) $$S_{PRO,D}=S_{PRO,\bar{D}}+S_{PRO,D^{\prime }}$$

## 4. Results and Discussion

### 4.1. Test Validation

**Figure 5:**
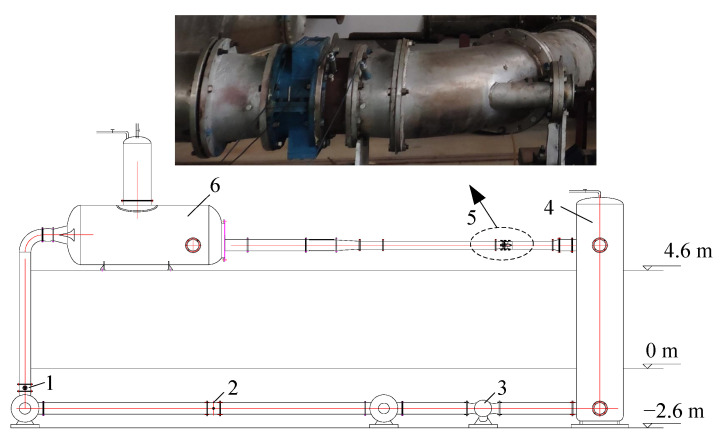
Structure of the test bench (1: butterfly damper, 2: electromagnetic flowmeter, 3: circulating pump, 4: high-pressure tank, 5: bidirectional axial-flow pump, 6: low-pressure tank).

To verify the accuracy of the numerical simulation, the external characteristics testing of an arc-shaped bidirectional axial-flow pump was completed. The test bench was a double-layered vertical structure, and the upper and lower heights were 4.6 m and −2.6 m, respectively, as shown in Figure 5. Low- and high-pressure tanks were located up- and downstream of the bidirectional axial-flow pump, respectively, to stabilize the water pressure. The electromagnetic flowmeter was located on the bottom layer to prevent interference from the bidirectional axial-flow pump. The specific parameters of the test instrument are shown in Table 2, and the measurement uncertainty of the system was calculated as less than 0.3% by $E_{S}=\sqrt{E_{Q}^{2}+E_{H}^{2}+E_{T}^{2}+E_{n}^{2}}$.

**Figure 6:**
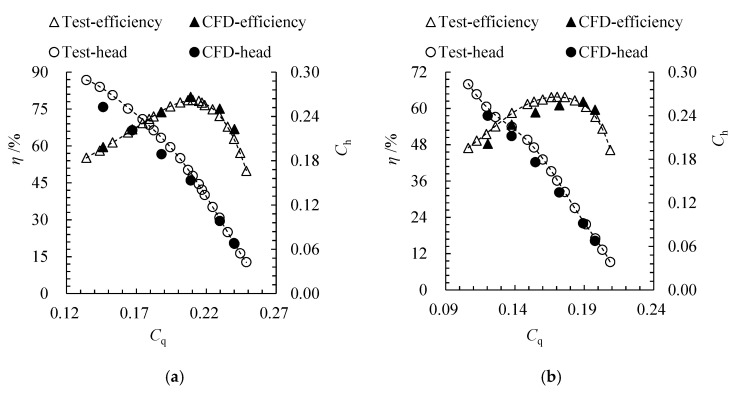
Comparison of hydraulic performance between the CFD results and the test data of the arc-shaped bidirectional axial-flow pump under (**a**) forward and (**b**) reverse conditions.

Figure 6 compares the hydraulic performance of the arc-shaped bidirectional pump based on the CFD results and test data. The head coefficient $C_{h}$ and flow coefficient $C_{q}$ can be calculated based on Equations (11)–(13). The calculated efficiency is slightly lower than the test efficiency under part-loaded conditions, but the calculated efficiency is slightly higher than the test efficiency under overloaded conditions. Under forward design flow rate, the relative error between the calculations and experiments of the efficiency and the head coefficient is 2.0% and −6.2%, respectively; under the reverse design point, those values are −4.3% and −8.5%, respectively.

(11) $$u_{2}=\frac{\pi d_{2}n}{60}$$

(12) $$C_{q}=\frac{Q}{\pi d_{2}(r_{s}-r_{h})u_{2}}$$

(13) $$C_{h}=\frac{2gH}{u_{2}^{2}}$$

where $u_{2}$ and n are the circumferential velocity and rotation speed, respectively, $d_{2}$ is the diameter of the impeller outlet, and $r_{s}$ and $r_{h}$ are the radii of the hub and the shroud, respectively.

### 4.2. Energy Characteristics of the Two Bidirectional Axial-Flow Pumps

**Figure 7:**
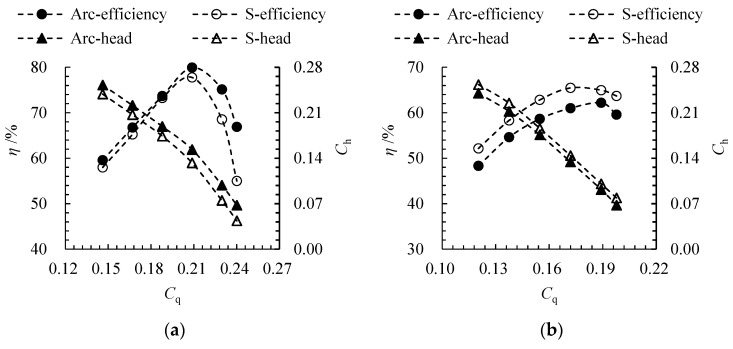
Comparison of pump performance between arc- and S-shaped cases under (**a**) forward and (**b**) reverse conditions.

Figure 7 compares the hydraulic performance of the arc- and S-shaped cases. Under the forward condition, the efficiency and head of the arc-shaped case are higher. The highest efficiencies of the arc- and S-shaped cases are 79.9% and 77.8% respectively, and the best efficiency point for both cases can be found at *C*_q_ = 0.21. The efficiency of the two cases is close—within *C*_q_ = 0.15–0.21—but the efficiency of the S-shaped case decreases sharply from *C*_q_ = 0.21 to *C*_q_ = 0.24, which indicates that the low-camber trailing edge of the S-shaped impeller leads to strong flow separation under a large flow coefficient. Under the reverse condition, the efficiency and head of the arc-shaped case are lower. The highest efficiencies of the arc- and S-shaped cases are 62.2% and 65.5%, respectively. The best efficiency points for the arc- and S-shaped cases can be seen at *C*′_q_ = 0.19 and *C*′_q_ = 0.17, respectively. The results indicate that the matching degree between the arc-shaped impeller and the inlet guide vanes is poor. The high-camber leading edge of the arc-shaped impeller decreases the attack angle of the inflow and, thus, increases the best efficiency point when compared with the S-shaped impeller.

**Figure 8:**
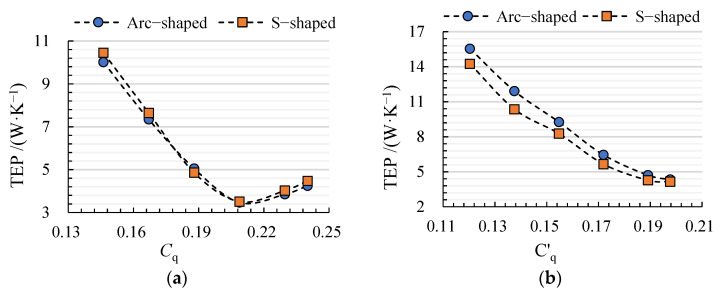
TEP distribution for arc- and S-shaped cases under (**a**) forward and (**b**) reverse conditions.

In this study, TEP is taken as the quantitative evaluation index of hydraulic loss, which is the key factor to determine the efficiency and head of an axial-flow pump. Figure 8 shows the TEP distribution with different flow coefficients for arc- and S-shaped cases. Under the forward condition, TEP first decreases and then increases with an increase in *C*_q_, and the minimum can be found under design condition *C*_q_ = 0.21, due to good matching between the impeller and the guide vanes. Except for *C*_q_ = 0.19, the TEP of S-shaped cases is higher than that of arc-shaped cases under all flow coefficients. The relative deviation between arc- and S-shaped cases is −1.12% under *C*_q_ = 0.21. The maximum relative deviation is −5.09%, which can be found under *C*_q_ = 0.24. Under the reverse condition, TEP decreases with a decline in *C*′_q_, which shows that an excessive attack angle of the impeller inflow due to inverted arch guide vanes is the main cause of hydraulic loss. The TEP of arc-shaped cases is higher than that of S-shaped cases under all flow coefficients, and the relative deviation between arc- and S-shaped cases is 13.95% under *C*′_q_ = 0.17 (design condition). The maximum relative deviation is 15.04%, which can be seen under *C*′_q_ = 0.14. Since the head and efficiency of axial-flow pumps decrease with an increase in hydraulic loss, the TEP curve can effectively express the influence of the impeller airfoil on the internal hydraulic loss, as shown in Figure 7 and Figure 8.

**Figure 9:**
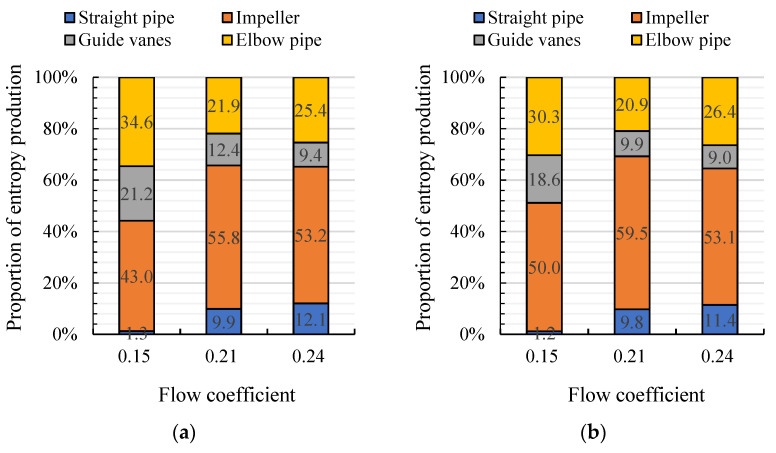
Proportion of entropy production of each hydraulic component for (**a**) arc-shaped and (**b**) S-shaped cases under the forward condition.

**Figure 10:**
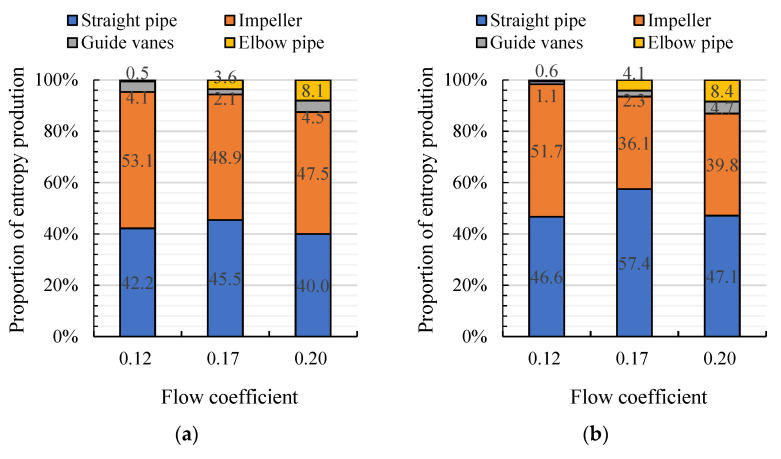
Proportion of entropy production of each hydraulic component for (**a**) arc-shaped and (**b**) S-shaped cases under the reverse condition.

Figure 9 shows the TEP proportion of each hydraulic component for arc- and S-shaped cases under three forward flow coefficients. The straight pipe is located upstream of the impeller, so there is no significant difference in TEP proportions between the two impeller cases. The TEP proportion of the impeller is obviously higher than that of the rest of the hydraulic components, showing that unstable flow inside the impeller is the main source of TEP. Under *C*_q_ = 0.15 and 0.21, the TEP proportion of the arc-shaped impeller is lower than that for the S-shaped impeller, showing that the unstable flow losses inside the S-shaped impeller lead to lower hydraulic performance. Under *C*_q_ = 0.24, the TEP proportion of each hydraulic component in the arc-shaped case is similar to that for the S-shaped case. The internal flow losses for the two impeller cases are approximately the same, but the lower blade outlet angle of the S-shaped impeller reduces the airfoil lift coefficient, and results in lower efficiency of the S-shaped impeller case. Figure 10 shows the TEP proportion of each hydraulic component for the arc- and S-shaped cases under three reverse flow coefficients. The TEP proportion of the straight pipe and impeller is obviously higher than that of the rest of the hydraulic components due to the distorted impeller inflow caused by an inverted arch guide vane. The TEP proportion of the arc-shaped impeller is higher than that of the S-shaped impeller, indicating that a higher blade inlet angle of the arc-shaped impeller leads to a strong impeller–guide vanes interference effect and, thus, hampers the pump efficiency.

### 4.3. Distribution of Local Entropy Production Rates under the Forward Condition

**Figure 11:**
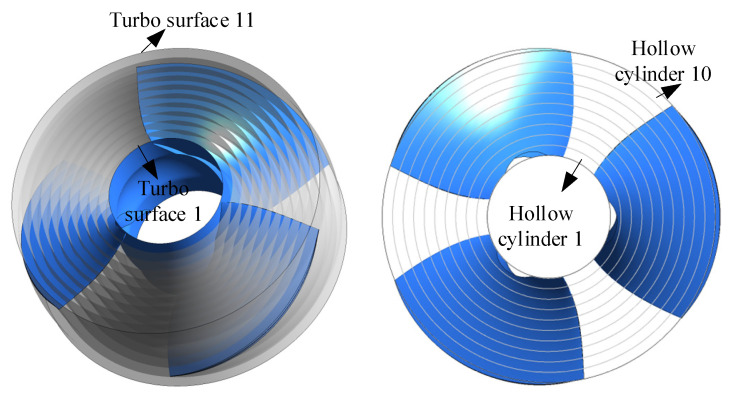
Position of the turbo surface and the volume.

The radial coefficient R^*^ of the turbo surface was defined as $\frac{r_{i}-r_{h}}{r_{s}-r_{h}}$, where $r_{i}$ is the radius of the calculated turbo surface. To quantitatively analyze the TEPR distribution in different sections of the impeller passage, the turbo surface *i* (*i* = 1, 2, 3…11) was used to divide the impeller passage evenly into 10 approximate hollow cylinders, as shown in Figure 11. The radial coefficient R* of the turbo surface *i* could be calculated by 0.1×i. The inner and outer walls of the hollow cylinder *i* are turbo surface *i* and turbo surface *i* + 1. 

**Figure 12:**
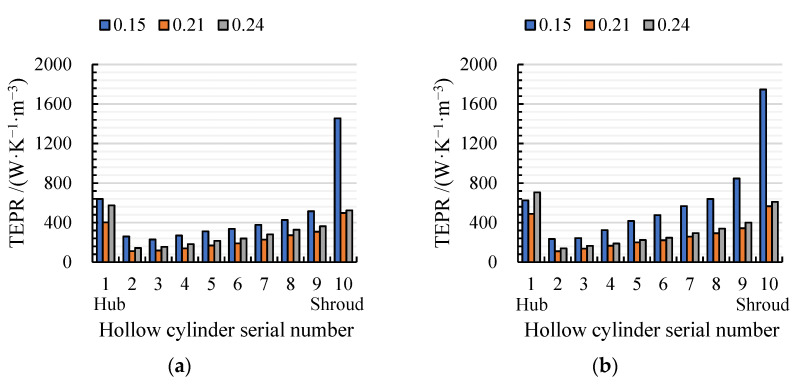
Volume average TEPR distribution in (**a**) arc-shaped and (**b**) S-shaped impellers under three forward flow coefficients.

**Figure 13:**
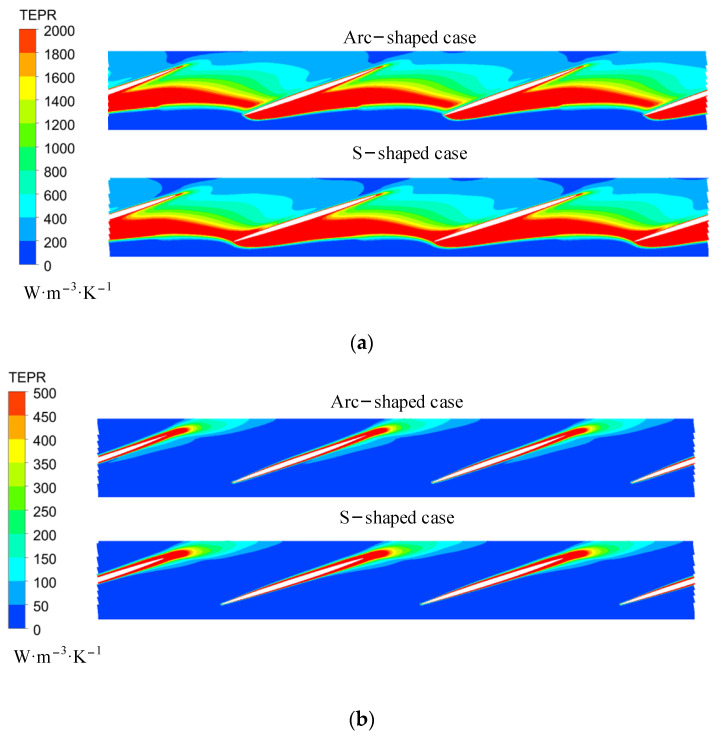

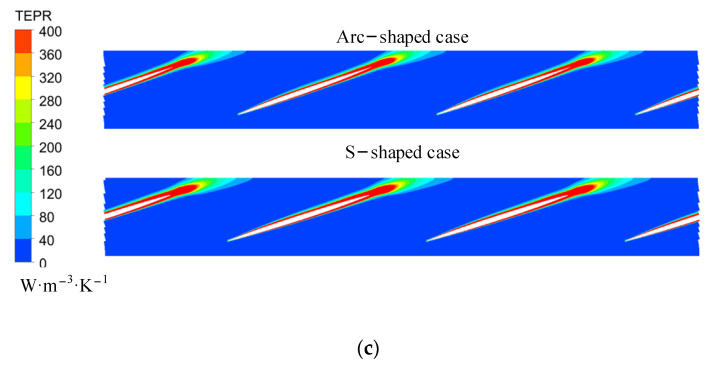
TEPR in the sections of the impeller passage under (**a**) *C*_q_ = 0.15, (**b**) *C*_q_ = 0.21, and (**c**) *C*_q_ = 0.24. (R* = 0.95).

Figure 12 shows the volume-averaged TEPR of 10 hollow cylinders in arc- and S-shaped impellers under the forward condition. The average TEPR of each volume is the highest and lowest under *C*_q_ = 0.15 and 0.21, respectively. Due to the wall effect flow, the average TEPR of the volumes first increased and then declined from hub to shroud. Compared with the arc-shaped impeller, the average TEPR for the S-shaped impeller was higher from volumes 5 to 9, which led to lower efficiency. In addition, the average TEPR of volumes 9 and 10 was obviously higher than those of the rest of the volumes, due to the tip clearance leakage flow. Accordingly, the spatial distribution of TEPR in the turbo surface near the impeller shroud (R* = 0.95) was obtained, as shown in Figure 13. Under *C*_q_ = 0.15, there was a large area of high TEPR near the leading edge due to strong flow separation caused by a large attack angle, and the TEPR gradually decreased from inlet to outlet. Under *C*_q_ = 0.21 and 0.24, the TEPR distributions of the two cases were similar, and there was no obvious high TEPR near the leading edge, since the attack angle decreased with an increase in the flow rate. However, mixing of the wake vortex and the main stream led to a small area of high TEPR near the trailing edge. In addition, the area of high TEPR near suction and the trailing edge of S-shaped blade case was slightly larger than that of the arc-shaped blade case, due to the anti-arch trailing edge of the S-shaped suction side.

**Figure 14:**
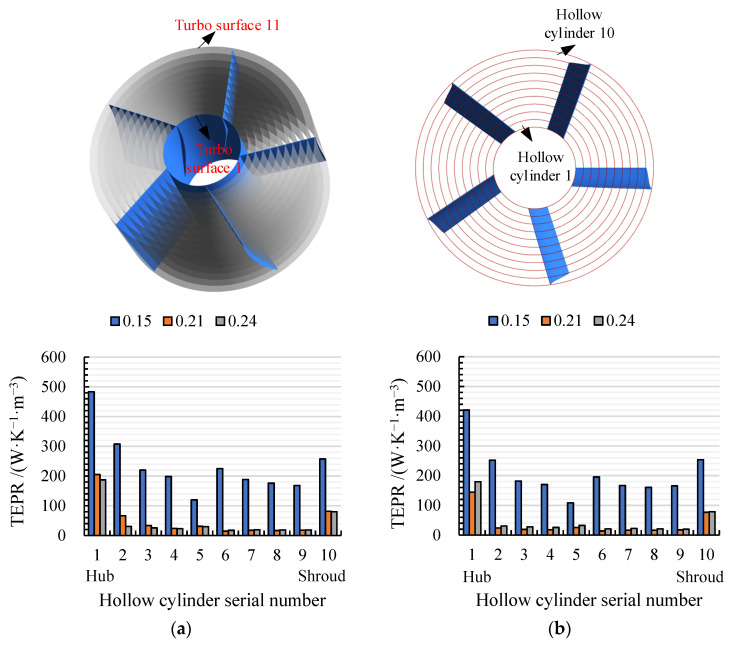
Volume-averaged TEPR distribution in guide vanes for (**a**) arc-shaped and (**b**) S-shaped cases under three forward flow coefficients.

To quantitatively analyze the TEPR of different sections under the forward condition, the passage of the guide vanes was also divided into 10 volumes. Figure 14 compares the volume-averaged TEPR of 10 hollow cylinders in guide vanes between the arc- and S-shaped impeller cases. The average TEPR decreased from volumes 1 to 9 due to the wall effect. Under *C*_q_ = 0.15, the average TEPR was extremely high, and the volume-averaged TEPR of the S-shaped impeller case was lower than that of the arc-shaped impeller case. In addition, the volume-averaged TEPR of the arc-shaped impeller case decreased with an increase in the flow rate, but the minimum for the S-shaped impeller case could be found under the design flow coefficient *C*_q_ = 0.21.

**Figure 15:**
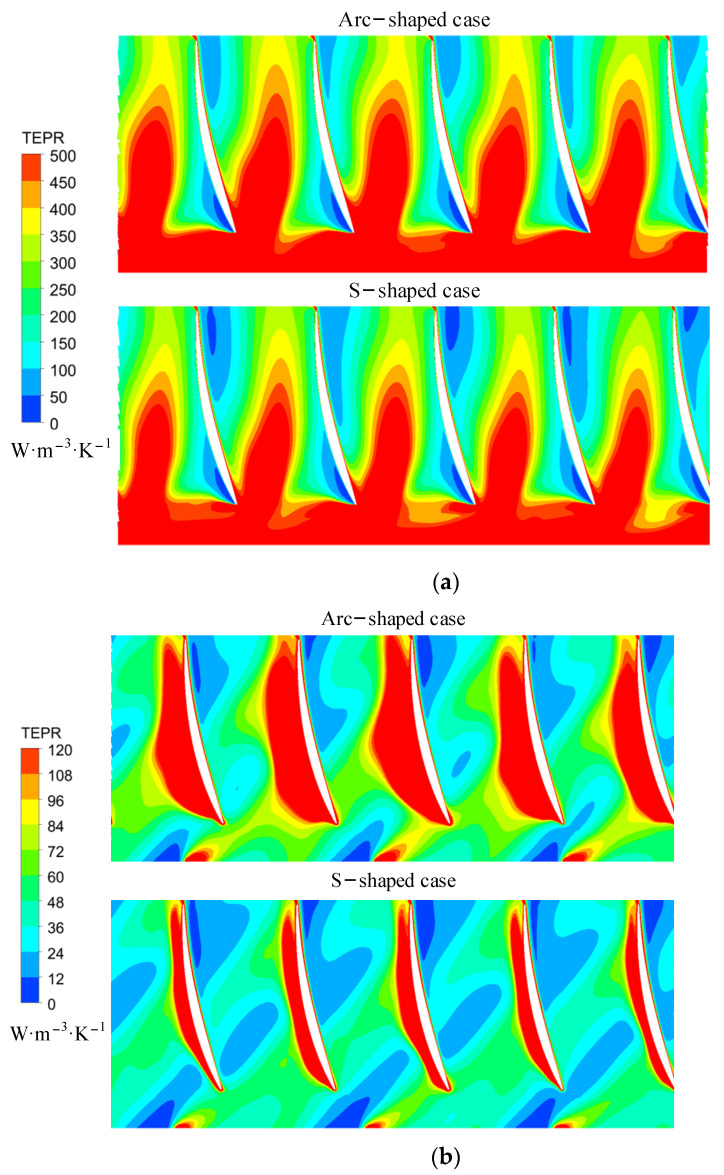

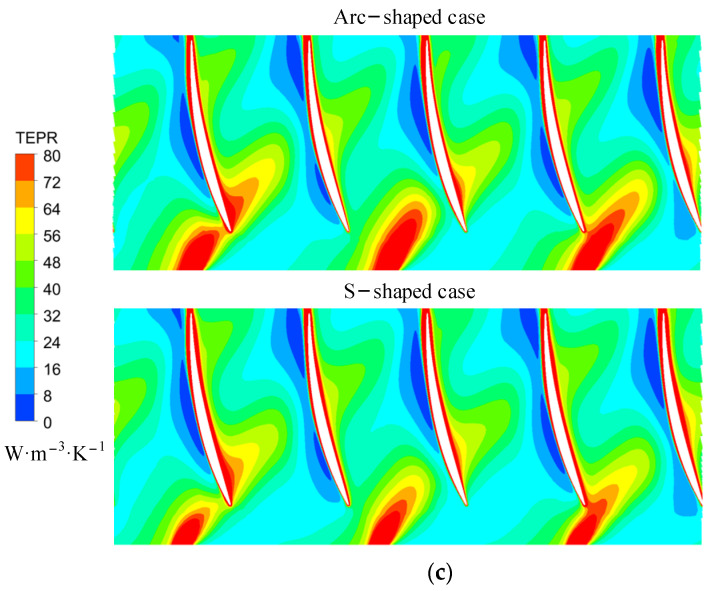
TEPR in the turbo surface of guide vanes under (**a**) *C*_q_ = 0.15, (**b**) *C*_q_ = 0.21, and (**c**) *C*_q_ = 0.24. (R* = 0.95).

Since the average TEPR of volume 1 was significantly higher than those of the rest of the volumes, this indicated that unstable flow structures were found near the hub. Thus, we determined the distribution of TEPR in the turbo surface with R* = 0.05, as shown in Figure 15. Due to interference between the impeller and the guide vanes, the TEPR near the inlet was high under all flow coefficients. However, the position of high TEPR near the surface of the guide vanes differed under the three flow coefficients. Under *C*_q_ = 0.15, the secondary vortex boundary near the leading edge was mixed with the main stream, resulting in high TEPR away from the suction side. Meanwhile, under *C*_q_ = 0.21 and 0.24, the internal fluid flowed along the surface of the guide vanes, meaning that the high TEPR could be found close to the surface of the guide vanes due to the wall effect.

**Figure 16:**
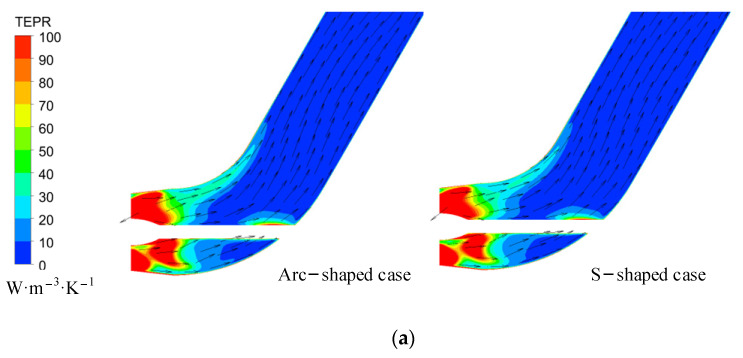

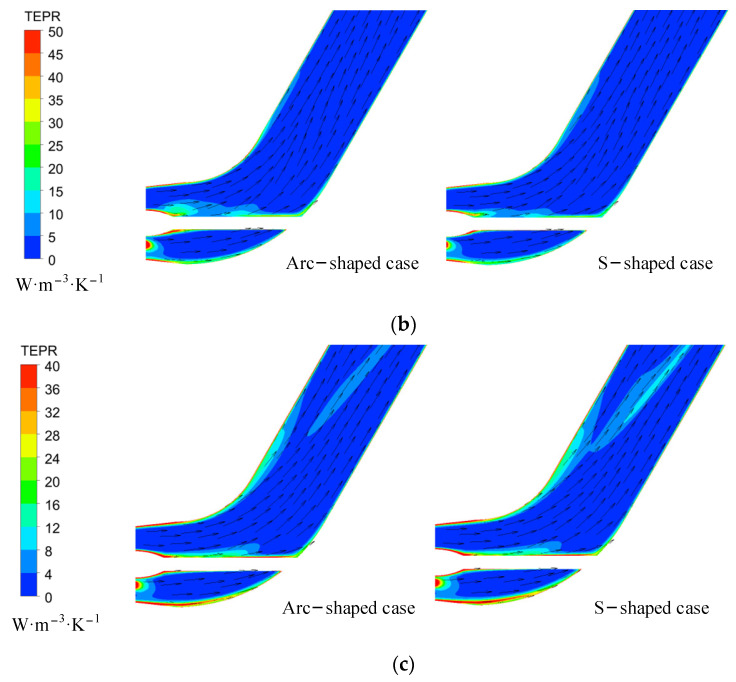
TEPR and velocity vector in the elbow pipe under (**a**) *C*_q_ = 0.15, (**b**) *C*_q_ = 0.21, and (**c**) *C*_q_ = 0.24. (R* = 0.95).

Figure 16 shows the distribution of TEPR in the elbow pipe under three forward flow coefficients. The TEPR distribution in the elbow pipe of S- and arc-shaped impeller cases was similar. There was high TEPR near the inlet, since the circulation of the internal flow gradually decreased in the flow direction, and the area of high TEPR decreased with an increase in the flow coefficients. Under *C*_q_ = 0.15, the backflow was upstream of the bend corner due to the high circumferential velocity, leading to high TEPR. Under *C*_q_ = 0.24, there was flow separation downstream of the bend corner due to the high axial velocity, which also resulted in high TEPR.

### 4.4. Distribution of Local Entropy Production Rate under the Reverse Condition

**Figure 17:**
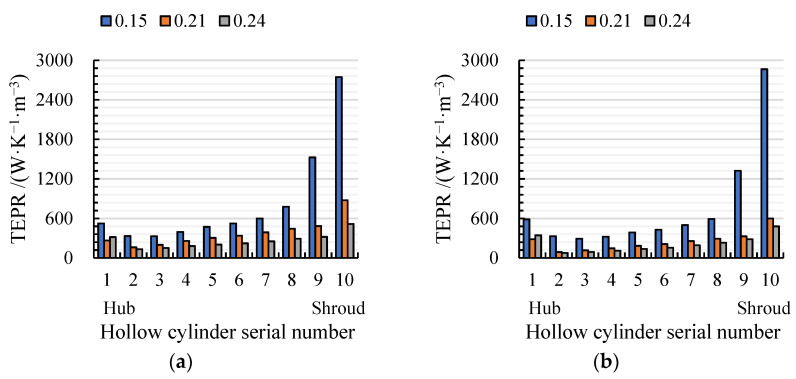
Volume-averaged TEPR distribution in (**a**) arc-shaped and (**b**) S-shaped impellers under three reverse flow coefficients.

**Figure 18:**
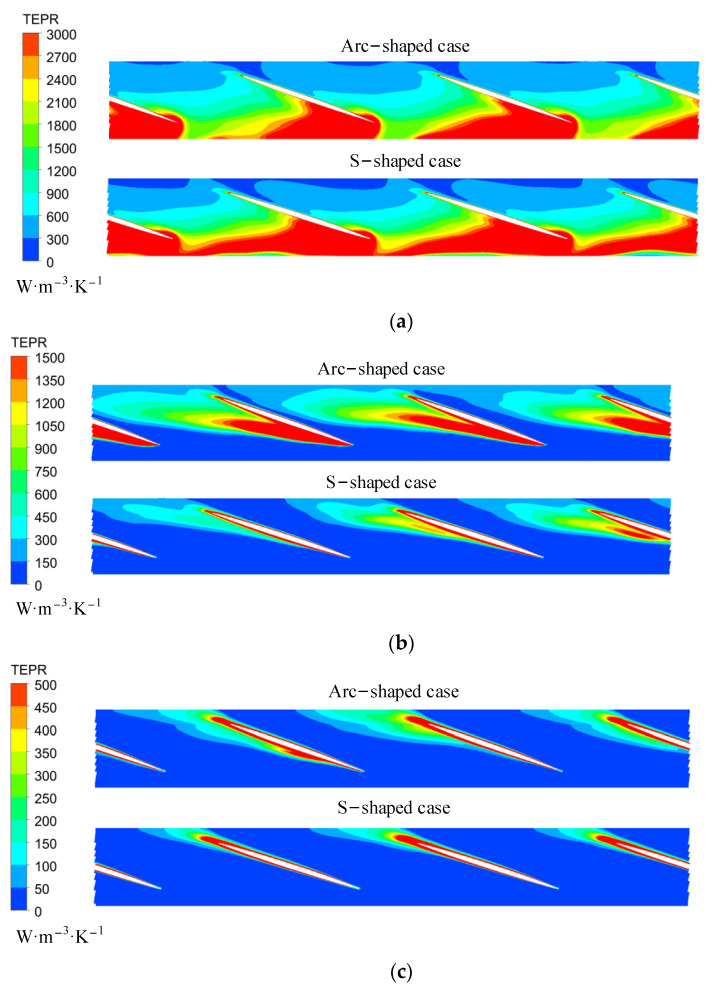
TEPR in the turbo surfaces of the impeller under (**a**) *C*′_q_ = 0.15, (**b**) *C*′_q_ = 0.21, and (**c**) *C*′_q_ = 0.24. (R* = 0.95).

Figure 17 shows the volume-averaged TEPR of 10 hollow cylinders in arc- and S-shaped impellers under the reverse condition, which is obviously higher than that under the forward condition, owing to the distorted impeller inflow. The average TEPR first decreases and then increases with an increase in the volume number due to the wall effect. Except for volume 1, the average TEPR of the rest of the volumes declines with an increase in the flow rate resulting from an improvement to the inflow pattern of the impeller. The average TEPR of each volume in the arc-shaped impeller is higher than that in the S-shaped impeller, which indicates that the large inlet angle of the arc-shaped blade leads to large, unstable flow structures. In addition, the average TEPR in volume 10 is obviously higher than those in the rest of the volumes, caused by tip clearance leakage flow. Given that, we determined the TEPR distribution in the turbo surface with R* = 0.95 of the arc- and S-shaped impellers under the reverse condition, as shown in Figure 18. Since the circumferential velocity decreases with an increase in the flow rate, the position of high TEPR caused by flow separation differs under the three flow coefficients. Under *C*′_q_= 0.15, the high TEPR can be found near the impeller inlet and the leading edge, due to backflow caused by the large circumferential velocity. Under *C*′_q_= 0.21, the high-TEPR area in both of the impellers shrinks significantly due to a reduction in the circumferential velocity. However, strong flow separation still exists at the leading edge of the arc-shaped impeller, resulting in a large area of high TEPR. Under *C*′_q_= 0.24, the flow pattern in both impellers is stable, and only a small area of high TEPR can be found near the trailing edge, caused by mixing of the wake vortices with the mainstream. Due to the larger inlet angle of the arc-shaped blade, the area of high TEPR near the suction and trailing edge is higher, compared with S-shaped blade. 

**Figure 19:**
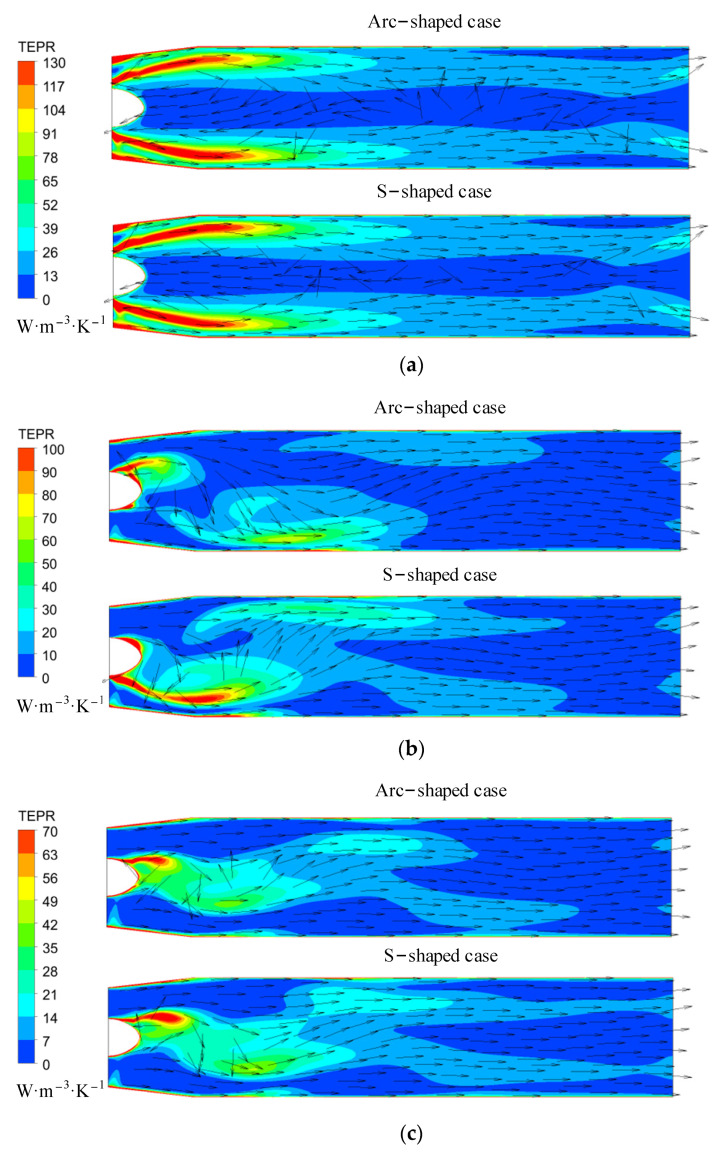
TEPR and velocity vector in the straight pipe under (**a**) *C*′_q_ = 0.15, (**b**) *C*′_q_ = 0.21, and (**c**) *C*′_q_ = 0.24.

Figure 19 shows the TEPR distribution in the straight pipe under the reverse condition. Since the internal TEPR of the straight pipe is mainly affected by the velocity circulation of the inflow, the internal TEPR decreases with an increase in the flow rate. High TEPR is located near the inlet, and decreases in the flow direction. According to Figure 7 and Figure 18, the velocity circulation of the inflow and the intensity of the wake vortex of the S-shaped impeller are higher and stronger than those of the arc-shaped impeller under the same flow coefficient. Thus, the area of high TEPR is larger in the S-shaped impeller case.

## 5. Conclusions

In this study, the URANS approach was used to predict the hydraulic performance and inner flow structure of bidirectional axial-flow pumps with arc- and S-shaped impellers. Entropy production theory was applied to evaluate the spatial distribution of internal hydraulic losses. The distribution of TEP and TEPR for the two impeller cases was similar, and the following conclusions can be drawn:

(1) Under the forward condition, the optimal operation point is not affected by the impeller airfoil, and the hydraulic performance of the arc-shaped case is higher than that of the S-shaped case. Under the reverse condition, the hydraulic performance is higher, and the optimal operation point shifts to a small flow rate of the S-shaped case, compared with the arc-shaped case.
(2) Under the forward condition, the TEP of the impeller and the elbow pipe is dominant. The anti-arch form of the trailing edge of the S-shaped suction side creates a barrier effect, which results in higher average TEPR near the region of the hub and the rim compared with the arc-shaped blade. In addition, the TEPR distribution in the elbow pipe is similar for the arc- and S-shaped cases, and the high TEPR can be found near inlet due to a decrease in the rotational kinetic energy along the flow direction.
(3) Under the reverse condition, the TEP of the impeller and the straight pipe is dominant. Due to the large inlet angle of the arc-shaped blade, the flow separation and TEPR near the suction side are stronger and higher, compared with the S-shaped case. Due to the stronger wake vortex, the area of high TEPR in the straight pipe of the arc-shaped case is higher than that of the S-shaped case.

These results provide useful suggestions for how to optimize the design of bidirectional axial-flow pumps.

## Figures and Tables

**Table 1:** Rated operational parameters of two bidirectional axial-flow pumps (calculated data).

Parameter	Unit	Arc-Shaped	S-Shaped
Forward-design flow rate	m/s	0.34	0.34
Forward-design head	m	3.3	2.9
Forward rotation speed	r/min	1350	1350
Forward specific speed		1173.5	1292.9
Reverse-design flow rate	m/s	0.28	0.28
Reverse-design head	m	2.9	3.1
Reverse rotation speed	r/min	1350	1350
Reverse specific speed		1626.0	1116.1

**Table 2:** Specific parameters of the test instruments.

MeasurementItems	Test Instrument	Measurement Range	Measurement Uncertainty
Flow rate	Electromagnetic flowmeter OPTIFLUX2000F	0~1800 m^3^/h	$E_{Q}^{}$ = 0.2%
Head	Intelligent differential pressure transmitter EJA	0~10 m	$E_{H}^{}$ = 0.1%
Torque	Intelligent torque speed sensor JCL1	0~200 N·m	$E_{T}^{}$ = 0.1%
Rotation speed			$E_{n}^{}$ = 0.1%

## Nomenclature

**Latin Letters**			
$\bar{p}$	Time-averaged pressure	S	Specific entropy
$\bar{v}$	Time-averaged velocity	T	Temperature
$\vec{q}$	Heat flux density vector	$C_{h}$	Head coefficient
$C_{q}$	Flow coefficient	$u_{2}$	Circumferential velocity
n	Rotation speed	*d*_2_	Diameter of impeller outlet
$r_{s}$	Hub radius	$r_{h}$	Shroud radius
*E*_Q_	Measurement uncertainty of flow rate	*E*_H_	Measurement uncertainty of head
*E*_T_	Measurement uncertainty of torque	*E*_n_	Measurement uncertainty of rotation speed
*R**	Radial coefficient	$S_{PRO,\bar{D}}$	Entropy production by direct dissipation
$S_{PRO,D^{\prime }}$	Entropy production by indirect dissipation	$S_{PRO,D}$	Total entropy production
**Greek Letters**			
ρ	Water density	μ	Dynamic viscosity
$\frac{\Phi }{T}$	Entropy production by dissipation	$\frac{\Phi_{\theta }}{T^{2}}$	Entropy production by heat transfer
$\frac{\Phi_{\bar{D}}}{\bar{T}}$	Entropy production rate by direct dissipation	$\frac{\Phi_{D^{\prime }}}{\bar{T}}$	Entropy production rate by indirect dissipation
**Abbreviations**			
URANS	Unsteady Reynolds time-averaged Navier–Stokes	CFD	Computational fluid dynamics
EXP	Experiment	TLV	Tip leakage vortex
TEP	Total entropy production	TEPR	Total entropy production rate
